# Mitochondrial dysfunction, cerebral metabolic crisis, and exploratory bioenergetic biomarkers in a translational swine model of acute carbon monoxide poisoning

**DOI:** 10.1093/toxsci/kfag093

**Published:** 2026-07-29

**Authors:** Abigail Insana, Alistair Lewis, John C Greenwood, Matthew Kelly, Frances S Shofer, Shih-Han Kao, McKenna Mason, Stephen Baak, Jonathan Starr, Jarelis Cabrera, Johannes K Ehinger, Todd J Kilbaugh, Tiffany Ko, Angela N Viaene, Wesley B Baker, David H Jang

**Affiliations:** Drexel University, Philadelphia, PA 19104, United States; Department of Neurology, Perelman School of Medicine, University of Pennsylvania, Philadelphia, PA 19104, United States; Division of Neurology, Children’s Hospital of Philadelphia, Philadelphia, PA 19104, United States; Department of Emergency Medicine, Perelman School of Medicine, University of Pennsylvania, Philadelphia, PA 19104, United States; Department of Emergency Medicine, Heersink School of Medicine, The University of Alabama at Birmingham, Birmingham, AL 35233, United States; Department of Emergency Medicine, Perelman School of Medicine, University of Pennsylvania, Philadelphia, PA 19104, United States; Resuscitation Science Center, The Children’s Hospital of Philadelphia, Philadelphia, PA 19104, United States; Department of Anesthesiology and Critical Care Medicine, Children’s Hospital of Philadelphia, Philadelphia, PA 19104, United States; Resuscitation Science Center, The Children’s Hospital of Philadelphia, Philadelphia, PA 19104, United States; Department of Anesthesiology and Critical Care Medicine, Children’s Hospital of Philadelphia, Philadelphia, PA 19104, United States; Resuscitation Science Center, The Children’s Hospital of Philadelphia, Philadelphia, PA 19104, United States; Department of Anesthesiology and Critical Care Medicine, Children’s Hospital of Philadelphia, Philadelphia, PA 19104, United States; Resuscitation Science Center, The Children’s Hospital of Philadelphia, Philadelphia, PA 19104, United States; Department of Anesthesiology and Critical Care Medicine, Children’s Hospital of Philadelphia, Philadelphia, PA 19104, United States; Resuscitation Science Center, The Children’s Hospital of Philadelphia, Philadelphia, PA 19104, United States; Department of Anesthesiology and Critical Care Medicine, Children’s Hospital of Philadelphia, Philadelphia, PA 19104, United States; Mitochondrial Medicine, Department of Clinical Sciences Lund, Lund University, Lund SE-221 84, Sweden; Otorhinolaryngology, Head and Neck Surgery, Department of Clinical Sciences Lund, Lund University, Lund SE-221 84, Sweden; Resuscitation Science Center, The Children’s Hospital of Philadelphia, Philadelphia, PA 19104, United States; Department of Anesthesiology and Critical Care Medicine, Children’s Hospital of Philadelphia, Philadelphia, PA 19104, United States; Department of Neurology, Perelman School of Medicine, University of Pennsylvania, Philadelphia, PA 19104, United States; Division of Neurology, Children’s Hospital of Philadelphia, Philadelphia, PA 19104, United States; Division of Neuropathology, Department of Pathology and Laboratory Medicine, Children’s Hospital of Philadelphia, Philadelphia, PA 19104, United States; Department of Pathology and Laboratory Medicine, Perelman School of Medicine at the University of Pennsylvania, Philadelphia, PA 19104, United States; Department of Neurology, Perelman School of Medicine, University of Pennsylvania, Philadelphia, PA 19104, United States; Division of Neurology, Children’s Hospital of Philadelphia, Philadelphia, PA 19104, United States; Department of Emergency Medicine, Perelman School of Medicine, University of Pennsylvania, Philadelphia, PA 19104, United States

**Keywords:** carbon monoxide poisoning, mitochondrial dysfunction, cerebral metabolism, cerebral microdialysis, bioenergetic biomarkers, swine model

## Abstract

Carbon monoxide (CO) poisoning remains a major cause of toxicologic morbidity and mortality and is a leading cause of acute neurologic injury among poisoned patients, yet the mechanisms underlying cerebral bioenergetic dysfunction remain incompletely understood. In addition to impaired oxygen delivery through carboxyhemoglobin (COHb) formation, CO poisoning is associated with mitochondrial respiratory dysfunction and cerebral metabolic injury. We characterized systemic physiology, cerebral metabolism, mitochondrial bioenergetics, and exploratory translational biomarkers in a swine model of acute CO poisoning. Yorkshire swine underwent sham exposure or inhalational CO exposure at 1,000 or 2,000 ppm with serial physiologic monitoring, arterial blood gas analysis, cerebral microdialysis, high-resolution mitochondrial respirometry, ATP quantification, western blotting, and histologic/immunohistochemical analyses. Peripheral blood mononuclear cell (PBMC) mitochondrial respiration was explored as a systemic correlate of cerebral mitochondrial function. CO exposure produced dose-dependent elevations in COHb and lactate with associated metabolic acidosis and hemodynamic impairment. Cerebral microdialysis demonstrated variable lactate-to-pyruvate ratios, whereas extracellular glycerol was significantly elevated during late exposure/recovery following severe CO exposure, consistent with membrane injury and metabolic dysfunction. Mitochondrial respiration was impaired in both cortical and hippocampal tissue, with Complex IV-linked respiration among the most consistently affected respiratory states. Cortical ATP content was significantly reduced in severely exposed animals, supporting cerebral bioenergetic failure. Western blot analysis demonstrated increased HO-1 expression without significant reductions in citrate synthase or Complex IV protein abundance, suggesting functional respiratory inhibition rather than loss of mitochondrial content. Collectively, these findings demonstrate that acute CO poisoning produces early cerebral bioenergetic dysfunction characterized by impaired mitochondrial respiration, ATP depletion, and metabolic alterations, while supporting the exploratory potential of PBMC mitochondrial respiration as a translational biomarker of cerebral mitochondrial dysfunction.

Carbon monoxide (CO) is a leading cause of environmental poisoning in the United States with substantial mortality and morbidity (Blumenthal 2001; Ran et al. 2018). More than 50,000 CO cases are evaluated in emergency departments annually (Raub et al. 2000; Long et al. 2021). Neurologic injury represents one of the most clinically significant consequences of acute CO poisoning, contributing to both immediate neurologic dysfunction and delayed neurocognitive sequelae among survivors (Weaver et al. 1996; Rose et al. 2018). Despite this clinical burden, objective biomarkers that reflect cerebral injury severity and recovery remain limited.

The pathophysiology of CO neurotoxicity is multifactorial and includes hypoxemia, impaired cellular oxygen utilization, neuroinflammation, and mitochondrial dysfunction (Jaffe 1997). A primary molecular target of CO is cytochrome *c* oxidase (Complex IV), which can impair oxidative phosphorylation and adenosine triphosphate (ATP) generation while promoting reactive oxygen species and downstream inflammatory cascades (Akyol et al. 2014; Chu et al. 2021). These mechanisms suggest that cerebral bioenergetic dysfunction may represent an early and potentially reversible stage of neurologic injury, highlighting the need for translational models capable of evaluating mitochondrial function and identifying clinically relevant biomarkers of cerebral metabolic dysfunction.

Current available biomarkers of cerebral injury include carboxyhemoglobin (COHb) and lactate levels in blood. Inhaled CO binds hemoglobin with substantially higher affinity than oxygen, forming COHb and reducing arterial oxygen content. Although COHb correlates with exposure severity and with nonspecific symptoms such as headache and dizziness, it does not reliably localize tissue injury or identify the underlying cellular mechanism (Ramirez et al. 1974). Serum lactate elevation reflects a systemic shift toward anaerobic metabolism but is similarly nonspecific for cerebral injury.

Peripheral blood mononuclear cells (PBMCs) have emerged as a potential minimally invasive biomarker of systemic mitochondrial dysfunction across multiple acute critical illnesses and toxicologic exposures (Jang et al. 2017a, 2017b, 2018a). Prior experimental and clinical work from our group demonstrated that PBMC mitochondrial respiration correlates with cerebral mitochondrial function following carbon monoxide poisoning and may better reflect organ-level bioenergetic injury than COHb alone (Jang et al. 2019b; Owiredu et al. 2020). In a prior rodent model of acute CO poisoning, increasing CO exposure produced dose-dependent reductions in mitochondrial respiratory states in both isolated brain mitochondria and PBMCs, supporting the translational application of blood-cell bioenergetics as a biomarker platform (Bungatavula et al. 2025).

Swine provide an important translational large-animal model for carbon monoxide poisoning because their cardiovascular physiology and cerebral anatomy more closely approximate those of humans than rodent models. This model enables the integration of advanced physiologic monitoring, cerebral microdialysis, high-resolution mitochondrial respirometry, tissue ATP quantification, and histopathologic assessment within a single experimental platform, providing a comprehensive evaluation of cerebral bioenergetic dysfunction that is not feasible in most preclinical models. In the current study, we leveraged this integrated translational platform to investigate the relationship between acute CO exposure, cerebral metabolism, and mitochondrial dysfunction using complementary physiologic, metabolic, and biomolecular assessments. We hypothesized that increasing CO exposure severity would produce progressive cerebral bioenergetic dysfunction characterized by impaired mitochondrial respiration, depletion of tissue ATP, and metabolic abnormalities preceding overt structural brain injury, while identifying translational biomarkers that may facilitate future diagnostics and therapeutic development.

## Materials and methods

### Animal model and study design

All procedures were approved by the Institutional Animal Care and Use Committee at the Children’s Hospital of Philadelphia and conducted in accordance with the National Institutes of Health Guide for the Care and Use of Laboratory Animals. Male (*n* = 8) and female (*n* = 7) Yorkshire pigs (∼10 kg and 3 to 5 wk of age) were used. Animals underwent baseline veterinary assessments and were acclimated for at least 24 h prior to experimentation. Premedication consisted of intramuscular ketamine (20 mg/kg), followed by induction with 5% isoflurane in 100% oxygen via snout mask to facilitate endotracheal intubation. After intubation, animals were transitioned to room air. Core temperature was monitored continuously throughout the experiment and maintained within the physiologic range using an external warming system as needed. Animals were randomly assigned to one of three groups using a computer-based randomization tool (Microsoft Excel): Sham (*n* = 5), CO-1000 (*n* = 5), and CO-2000 (*n* = 5). This developmental stage corresponds to an early juvenile large-animal model with ongoing postnatal cerebral maturation, which may influence mitochondrial function, oxidative stress responses, and susceptibility to hypoxic or toxic injury (Helke and Swindle 2013; Tohyama and Kobayashi 2019).

### Perioperative monitoring and instrumentation

Animals were mechanically ventilated (tidal volume 10 to 11 ml/kg; PEEP 5 cm H_2_O; respiratory rate adjusted to maintain end-tidal CO_2_ 38 to 42 mmHg). Femoral arterial and venous access were established for continuous monitoring and sampling. Isoflurane was reduced to 0.5% to 1% to minimize confounding effects on mitochondrial function. Sedation during exposure was maintained with intravenous fentanyl (5 µg/kg/h) and dexmedetomidine (2 µg/kg/h). Core temperature was monitored continuously. Physiologic data were acquired using PowerLab 16/35 with LabChart 8 Pro software (ADInstruments, Colorado Springs, CO, United States).

### Carbon monoxide exposure protocol

CO was delivered at 1,000 or 2,000 ppm using a calibrated gas delivery system (Airgas, Radnor, PA, United States) with a fraction of inspired oxygen (FiO_2_) of 0.21. Inspired CO concentrations were continuously verified at the endotracheal tube using a handheld electrochemical CO detector (Sensorcon Inspector, model INS-CO-01; Sensorcon, Arden, NC, United States). Animals were exposed to CO for 180 min, followed by 30 min of room air (total experimental duration 210 min). Sham animals received room air throughout. Systemic CO exposure was assessed using continuous pulse CO-oximetry (Rad-57, Masimo Corporation, Irvine, CA, United States) and serial venous blood gas (VBG) measurements for direct quantification of COHb. This multimodal approach enabled verification of delivered, inspired, and systemic CO exposure.

### Brain tissue collection and mitochondrial isolation

Following euthanasia with potassium chloride, brains were rapidly extracted and sectioned coronally. Brain harvest and tissue snap-freezing were performed using a standardized protocol with minimized postmortem interval across groups. Cortical and hippocampal regions were isolated. Mitochondria were isolated separately from cortical and hippocampal tissue using identical protocols of differential centrifugation with a discontinuous Percoll density gradient, as adapted from established protocols. All procedures were performed at 4°C with pre-chilled buffers and equipment to preserve mitochondrial integrity. Fresh brain tissue was rinsed and minced on ice, then homogenized in isolation buffer (320 mM sucrose, 2 mM EGTA, 10 mM Tris–HCl, pH 7.4) supplemented with fatty acid-free bovine serum albumin. Homogenization was performed using a glass Dounce homogenizer with sequential loose and tight pestle strokes to achieve a uniform suspension while minimizing shear-induced mitochondrial damage. The homogenate was subjected to low-speed centrifugation (1,300 × *g*, 3 min) to remove cellular debris and nuclei. The resulting supernatant was collected, re-extracted once, and combined prior to high-speed centrifugation (21,000 × *g*, 10 min) to pellet crude mitochondria. The mitochondrial pellet was resuspended in 15% Percoll and layered onto a discontinuous Percoll gradient (15%, 23%, and 40%) prepared in isolation buffer. Following ultracentrifugation (30,000 × *g*, 10 min, slow acceleration, no brake), nonsynaptosomal mitochondria were collected from the interface above the 40% layer, avoiding contamination from myelin and synaptosomal fractions. Isolated mitochondria were washed twice in isolation buffer to remove Percoll (16,700 × *g* and 6,900 × *g* sequential spins) and gently resuspended for downstream analyses and finally protein concentration was determined using a bicinchoninic acid assay based on our prior works (Jang et al. 2021; Lewis et al. 2022).

### Blood collection and PBMC isolation and analysis

Whole blood was collected into EDTA tubes at predefined study time points (baseline, end of carbon monoxide exposure, and end of re-oxygenation) for plasma collection and downstream biomarker analyses. In a subset of animals, an additional whole blood sample collected at the end of the exposure period was processed for peripheral blood mononuclear cell (PBMC) isolation to evaluate PBMC mitochondrial respiration as an exploratory translational biomarker of cerebral mitochondrial dysfunction. PBMCs were isolated by density gradient centrifugation using Ficoll-Paque. Briefly, whole blood was diluted with phosphate-buffered saline (PBS), layered over Ficoll-Paque, and centrifuged to isolate the PBMC fraction. Cells were subsequently washed with PBS, counted, and prepared for high-resolution respirometry. Prior to mitochondrial respiration measurements, PBMCs were permeabilized with digitonin to permit substrate access to the mitochondria. Mitochondrial respiratory function was then assessed using the same substrate–uncoupler–inhibitor titration (SUIT) protocol described for isolated brain mitochondria, as previously reported by our group. (Jang et al. 2018b, 2019a).

### Mitochondrial respiration (high-resolution respirometry)

Mitochondrial respiratory function was assessed in isolated cortical and hippocampal mitochondria using high-resolution respirometry (Oroboros O2k, Oroboros Instruments, Innsbruck, Austria) according to a standardized substrate–uncoupler–inhibitor titration (SUIT) protocol. Sequential additions of respiratory substrates, ADP, uncouplers, and inhibitors were used to quantify oxygen consumption across multiple respiratory states, including LEAK respiration (LEAK_PM_), oxidative phosphorylation supported by Complex I (OXPHOS_CI_) and convergent Complex I + II substrates (OXPHOS_CI + CII_), uncoupled electron transport system capacity supported by Complex I + II (ETS_CI + CII_) and Complex II (ETS_CII_), and Complex IV (CIV)-linked respiration.

Respiration was normalized to mitochondrial protein concentration and expressed as oxygen flux (pmol O_2_·s^−1^·mg protein^−1^). Cortical and hippocampal mitochondria were analyzed separately. Group differences across respiratory states were evaluated using mixed-effects models with respiratory state as the repeated measure and treatment group as the between-subject factor. When significant main effects or interactions were identified, post hoc multiple-comparison testing was performed to identify differences between exposure groups within individual respiratory states (version 7, Oroboros Instruments). (Pesta and Gnaiger 2012; Miller et al. 2017).

### Cerebral microdialysis

Cerebral microdialysis probes (CMA 70 Elite, mDialysis, Stockholm, Sweden) were placed in the left frontal cortex at a depth of ∼0.5 to 1 cm under sterile conditions. Probes were connected to a microinfusion pump (CMA 106, mDialysis) and perfused with sterile normal saline at a constant flow rate of 1 µL/min. Prior to sample collection, probes were allowed to equilibrate for 30 min to ensure stable baseline conditions, during which dialysate was discarded. Following equilibration, dialysate samples were collected at 30-min intervals into microvials and immediately stored at −80°C until analysis. Metabolite concentrations, including lactate, pyruvate, glucose, glycerol, and glutamate, were quantified using an ISCUS Flex analyzer (mDialysis, Stockholm, Sweden). The lactate-to-pyruvate ratio (LPR) was calculated as an index of cerebral redox state and mitochondrial function, whereas glycerol levels were used as a marker of cellular membrane breakdown and injury.

### ATP quantification

ATP concentrations were measured in cortical brain tissue using a fluorometric assay (ATP Assay Kit, Abcam, ab83355, Cambridge, MA, United States). Given the labile nature of ATP, tissue samples were rapidly harvested, snap-frozen in liquid nitrogen, and stored at −80°C until analysis. For quantification, tissue samples were homogenized in ATP assay buffer at a ratio of ∼10 mg tissue per 100 µL buffer under cold conditions to minimize enzymatic ATP degradation. Homogenates were centrifuged as needed to remove debris, and supernatants were used for analysis. Samples were loaded in duplicate into opaque 96-well plates, with input volumes adjusted (2 to 50 µL) and brought to a final volume of 50 µL with assay buffer. Where appropriate, samples were diluted to fall within the linear range of detection. A reaction mix containing ATP probe, converter, and developer was added to each well, followed by incubation at room temperature for 30 min protected from light. Fluorescence was measured (Ex/Em: 535/587 nm), and ATP concentrations were calculated from standard curves generated using serial dilutions of ATP standards. Background signal was corrected using sample blanks lacking ATP converter to account for nonspecific signal. ATP concentrations were normalized to tissue input and expressed as [nmol/mg tissue].

### Multiplex cytokine analysis (quansys platform)

Cytokine concentrations were measured using a multiplex chemiluminescent immunoassay platform (Q-Plex, Quansys Biosciences, Logan, UT, United States). Plasma samples were stored at −80°C and thawed on ice prior to analysis. Samples and calibrators were prepared in duplicate using a separate loading plate, followed by transfer to the Q-Plex array plate (50 µL per well), taking care to avoid contact with the antibody-printed surface. Calibrators were generated via serial dilution to construct standard curves spanning the dynamic range of the assay. Following sample loading, plates were incubated with shaking for 3 h at room temperature to allow binding to immobilized capture antibodies. Plates were then washed using an automated plate washer to remove unbound material. Detection was performed sequential incubation with biotinylated detection antibodies (90 min) and streptavidin–horseradish peroxidase (20 min), with washing steps between each stage. Chemiluminescent substrate (Substrate A/B mixture) was added, and signal was immediately detected using a Q-Plex imager. Image acquisition parameters were standardized across plates. Cytokine concentrations were calculated using standard curves generated from calibrators and analyzed with Q-View software (Quansys Biosciences), incorporating correction for sample dilution and background signal.

### Determination of protein expression

Snap-frozen cortical brain tissue was homogenized in ice-cold RIPA buffer supplemented with protease inhibitors, briefly sonicated, and centrifuged (14,000 × *g*, 10 min, 4°C) to remove insoluble material. Protein concentration in the supernatant was quantified using a bicinchoninic acid (BCA) assay. Equal amounts of protein were separated by SDS–PAGE on 4% to 12% Bis–Tris gels and transferred to nitrocellulose. Membranes were blocked and incubated with primary antibodies against citrate synthase (CS), heme oxygenase-1 (HO-1), and Complex IV (COX IV), with GAPDH or GA1R used for normalization. Following incubation with species-appropriate HRP-conjugated secondary antibodies, bands were visualized by chemiluminescence. Densitometric analysis was performed using iBright Analysis Software with local background correction, and target protein expression was normalized to the loading control. Each sample was analyzed using duplicate lanes across two independent immunoblots where sufficient tissue was available. Technical replicate densitometry measurements were averaged to generate a single value for each biological replicate prior to statistical analysis.

### Histopathology

Following completion of exposure and euthanasia, cortical and hippocampal brain tissue was harvested, fixed in 10% neutral buffered formalin, paraffin-embedded, and sectioned at 6 μm. For each animal, one representative section containing the cortex and hippocampus were analyzed. Sections were deparaffinized, rehydrated, and subjected to heat-induced antigen retrieval in sodium citrate buffer (pH ∼6.0) using a pressure-based system, followed by cooling and PBS washes. Endogenous peroxidase activity was quenched, and nonspecific binding was blocked prior to overnight incubation at 4°C with primary antibodies against citrate synthase (CS), Complex IV (COXIV), IBA1 (microglial activation), GFAP (astroglial reactivity), and PARP. Sections were subsequently incubated with the appropriate secondary antibodies, developed using diaminobenzidine (DAB), counterstained with hematoxylin, dehydrated, and coverslipped.

Histopathologic and immunohistochemical evaluation was performed by a pathologist (A.V.) who was blinded to treatment assignment using a semiquantitative ordinal scoring system (0–3) where 0 indicated no staining and 3 indicated prominent pathological staining. Scores were assigned separately for the cortex and hippocampus for each stain and summarized by treatment group. Representative images were selected from sections demonstrating staining patterns representative of the assigned semiquantitative scores.

### Statistical analysis

Continuous variables are reported as mean ± SD or median with interquartile range (IQR), as appropriate, based on distributional characteristics assessed using the Shapiro–Wilk test. Given the small per-group sample sizes, distributional testing was interpreted cautiously, and nonparametric methods were prioritized when normality assumptions could not be confirmed. Categorical variables are presented as counts and proportions.

Between-group comparisons were performed using Student’s *t*-test or one-way analysis of variance (ANOVA) for parametric data and Mann–Whitney *U* or Kruskal–Wallis tests for nonparametric data, as appropriate. When omnibus group differences were significant, post hoc multiple-comparison testing was performed using Tukey’s test following one-way ANOVA or Dunn’s test with Holm adjustment following Kruskal–Wallis analysis.

Mitochondrial respiratory data and serial cerebral microdialysis measurements were analyzed using linear mixed-effects models with restricted maximum likelihood (REML) estimation, incorporating fixed effects of respiratory state or time, treatment group, and their interaction, with animal included as a random effect to account for repeated within-subject measurements. Post hoc multiple-comparison testing for mixed-effects models was performed using Tukey’s correction.

Linear regression was used to assess relationships between lactate-to-pyruvate ratio and physiologic variables. Correlations between PBMC and tissue mitochondrial endpoints were analyzed using Pearson or Spearman correlation coefficients, as appropriate, and are reported as exploratory analyses without formal correction for multiple comparisons given the limited number of prespecified pairings.

For one-way ANOVA and mixed-effects analyses, F statistics and corresponding *P*-values are reported where appropriate. A two-sided *P *< 0.05 was considered statistically significant. Statistical analyses were performed using SAS (v15.1) and GraphPad Prism (v9.0).

## Results

### Cohort characteristics and exposure verification

Physiologic and metabolic responses following carbon monoxide exposure are summarized in Table 1. Compared with sham animals, CO exposure produced dose-dependent systemic toxicity characterized by progressive increases in carboxyhemoglobin saturation and blood lactate concentrations with concomitant reductions in arterial pH and bicarbonate. Animals exposed to 2,000 ppm CO also demonstrated lower diastolic blood pressure and mean arterial pressure at the terminal timepoint, consistent with more severe hemodynamic compromise.

**Table 1.** Terminal hemodynamic and arterial blood gas parameters following carbon monoxide exposure.

Parameter	Sham *(N = 5)*	CO-1000 *(N = 5)*	CO-2000 *(N = 3)*	Overall *P*-value
Hemodynamics				
Heart rate, bpm	122.1 ± 9.8	159.4 ± 32.5	113.4 ± 45.4	0.154
Systolic BP, mmHg	72.2 ± 29.7	69.5 ± 6.0	33.8 ± 27.4	0.104
Diastolic BP, mmHg	43.2 ± 17.4	39.6 ± 4.8	18.5 ± 9.1^†^	0.011
MAP, mmHg	56.1 ± 20.4	53.7 ± 5.0	25.2 ± 18.4^†^	0.033
Arterial blood gas/metabolic parameters				
pH	7.52 ± 0.03	7.47 ± 0.07	7.33 ± 0.03^†^	0.023
pCO_2_, mmHg	36.7 ± 2.3	36.2 ± 2.9	34.3 ± 2.2	0.313
pO_2_, mmHg	87.1 ± 6.8	105.3 ± 14.1	98.5 ± 7.7	0.185
Lactate, mmol/l	0.8 ± 0.2	3.1 ± 2.0	8.3 ± 3.5^†^^‡^	0.022
COHb saturation, %	0.9 ± 0.9	52.8 ± 1.4^†^	68.0 ± 1.6^†^^‡^	0.030
Bicarbonate, mmol/l	29.7 ± 1.1	26.4 ± 3.0	18.1 ± 1.3^†^^‡^	0.020

Data are presented as mean ± SD of terminal measurements obtained immediately prior to euthanasia. Overall *P*-values were calculated using the Kruskal–Wallis test. Pairwise comparisons were performed using Dunn’s test with Holm adjustment for multiple comparisons.

† *P* < 0.05 vs Sham.

‡ *P* < 0.05 vs CO-1000.

CO-2000 values represent animals surviving to the terminal study time point (*N* = 3).

Bpm, beats per minute; BP, blood pressure; MAP, mean arterial pressure; COHb, carboxyhemoglobin.

### Mitochondrial respiratory dysfunction

**Fig. 1.**
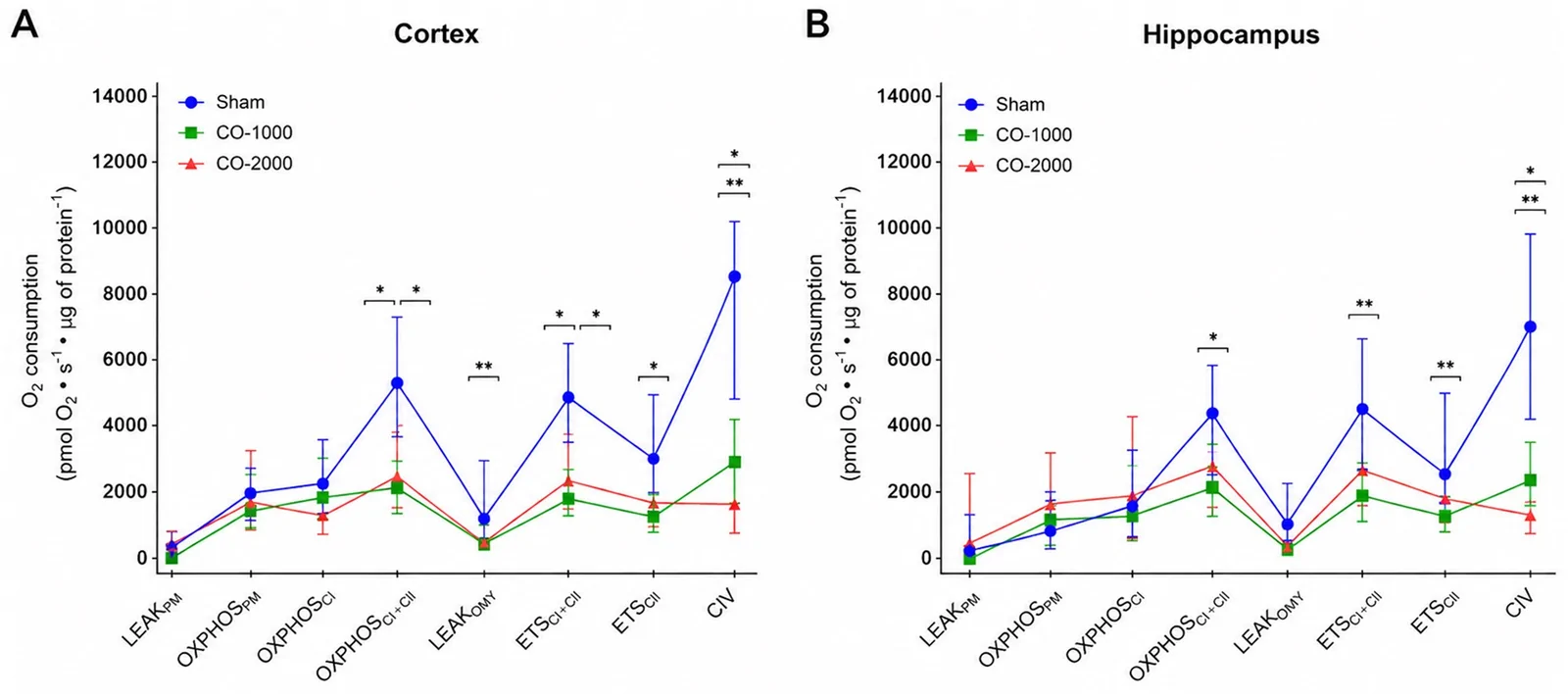
Acute carbon monoxide exposure impairs mitochondrial respiration in cortex and hippocampus. High-resolution respirometry was performed on isolated mitochondria from the (A) cortex and (B) hippocampus following sham exposure, CO-1000, or CO-2000. Respiratory states included pyruvate/malate-supported LEAK respiration (LEAK_PM_), ADP-stimulated oxidative phosphorylation supported by pyruvate and malate (OXPHOS_PM_), Complex I-supported oxidative phosphorylation following glutamate addition (OXPHOS_CI_), convergent Complex I + II-supported oxidative phosphorylation (OXPHOS_CI + CII_), oligomycin-induced LEAK respiration (LEAK_Omy_), uncoupled Complex I + II electron transport system capacity following FCCP titration (ETS_CI + CII_), uncoupled Complex II-supported electron transport system capacity following rotenone inhibition (ETS_CII_), and Complex IV-linked respiration (CIV). Data are presented as median with interquartile range. (Sham, *n* = 5; CO-1000, *n* = 5; CO-2000, *n* = 3). Mixed-effects analysis demonstrated significant effects of respiratory state, exposure group, and respiratory state × exposure interaction in both brain regions. Acute CO exposure reduced mitochondrial respiration across multiple respiratory states, with Complex IV-linked respiration representing one of the most consistently affected respiratory states in both cortex and hippocampus. Respiratory states are displayed in the sequential order of the Oroboros substrate–uncoupler–inhibitor titration (SUIT) protocol; connecting lines are included to illustrate the overall mitochondrial respiratory profile across ordered respiratory states and do not represent temporal measurements. Statistical significance is denoted as follows: *P *< 0.05 (*); *P *< 0.01 (**).

Mitochondrial respiratory function was evaluated in isolated cortical and hippocampal mitochondria using high-resolution respirometry across sequential substrate–uncoupler–inhibitor titration states (Fig. 1). Mixed-effects analysis demonstrated significant effects of respiratory state, exposure group, and respiratory state × exposure interaction in both cortex and hippocampus, indicating that the impact of CO exposure differed across mitochondrial respiratory states.

In cortical tissue, sham animals demonstrated higher oxygen flux than CO-exposed groups across most respiratory states. Significant reductions were observed in convergent Complex I and II-supported oxidative phosphorylation (OXPHOS_CI + CII_), uncoupled Complex I and II electron transport system capacity (ETS_CI + CII_), uncoupled Complex II-supported respiration (ETS_CII_), and Complex IV-linked respiration (CIV) in CO-exposed animals compared with sham controls. Oligomycin-induced LEAK respiration (LEAK_Omy_) was also reduced in the CO-2000 group. Among the cortical respiratory states, CIV-linked respiration demonstrated one of the largest reductions relative to sham animals.

In hippocampal tissue, CO exposure also resulted in significant reductions in LEAK_Omy_ respiration and CIV-linked respiration compared with sham animals. Additional numerical reductions were observed across OXPHOS and ETS respiratory states, including OXPHOS_CI + CII_, ETS_CI + CII_, and ETS_CII_; however, these comparisons did not consistently remain significant following correction for multiple comparisons. As in the cortex, CIV-linked respiration was among the most consistently affected respiratory states following CO exposure.

Across both brain regions, sham animals consistently demonstrated the highest respiratory capacity, whereas CO exposure produced broad suppression of mitochondrial oxygen consumption across multiple respiratory states. Collectively, these findings demonstrate impaired mitochondrial bioenergetics following acute carbon monoxide poisoning, with Complex IV-linked respiration emerging as one of the most consistently affected respiratory states in both cortical and hippocampal tissue.

### ATP depletion following CO exposure

**Fig. 2.**
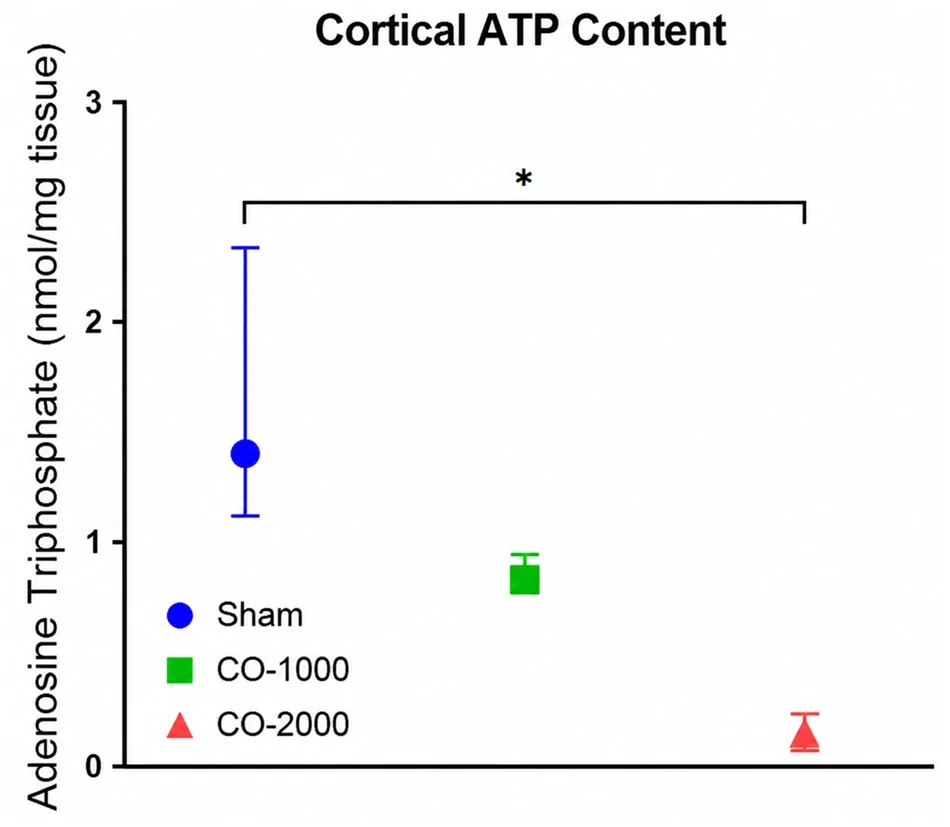
Acute carbon monoxide exposure reduces cortical ATP content. ATP concentrations were measured in cortical brain tissue harvested following sham exposure, CO-1000, or CO-2000 using a fluorometric ATP assay. Data are presented as median with interquartile range and expressed as nmol/mg tissue. Sham, *n* = 5; CO-1000, *n* = 5; CO-2000, *n* = 3). Cortical ATP content was significantly reduced in the CO-2000 group compared with sham (*P* < 0.05). ATP levels in the CO-1000 group were intermediate and were not significantly different from sham.

ATP content was measured in cortical brain tissue following sham exposure, 1,000 ppm, and 2,000 ppm CO exposure (Fig. 2). Sham animals demonstrated the highest ATP levels, whereas animals exposed to 2,000 ppm exhibited a marked reduction in ATP content. Cortical ATP levels were significantly lower in the CO-2,000 group compared with sham (*P *< 0.05). ATP values in the CO-1,000 group were intermediate between sham and CO-2,000 group but did not differ significantly from sham.

### PBMC mitochondrial respiration correlation analysis

**Fig. 3.**
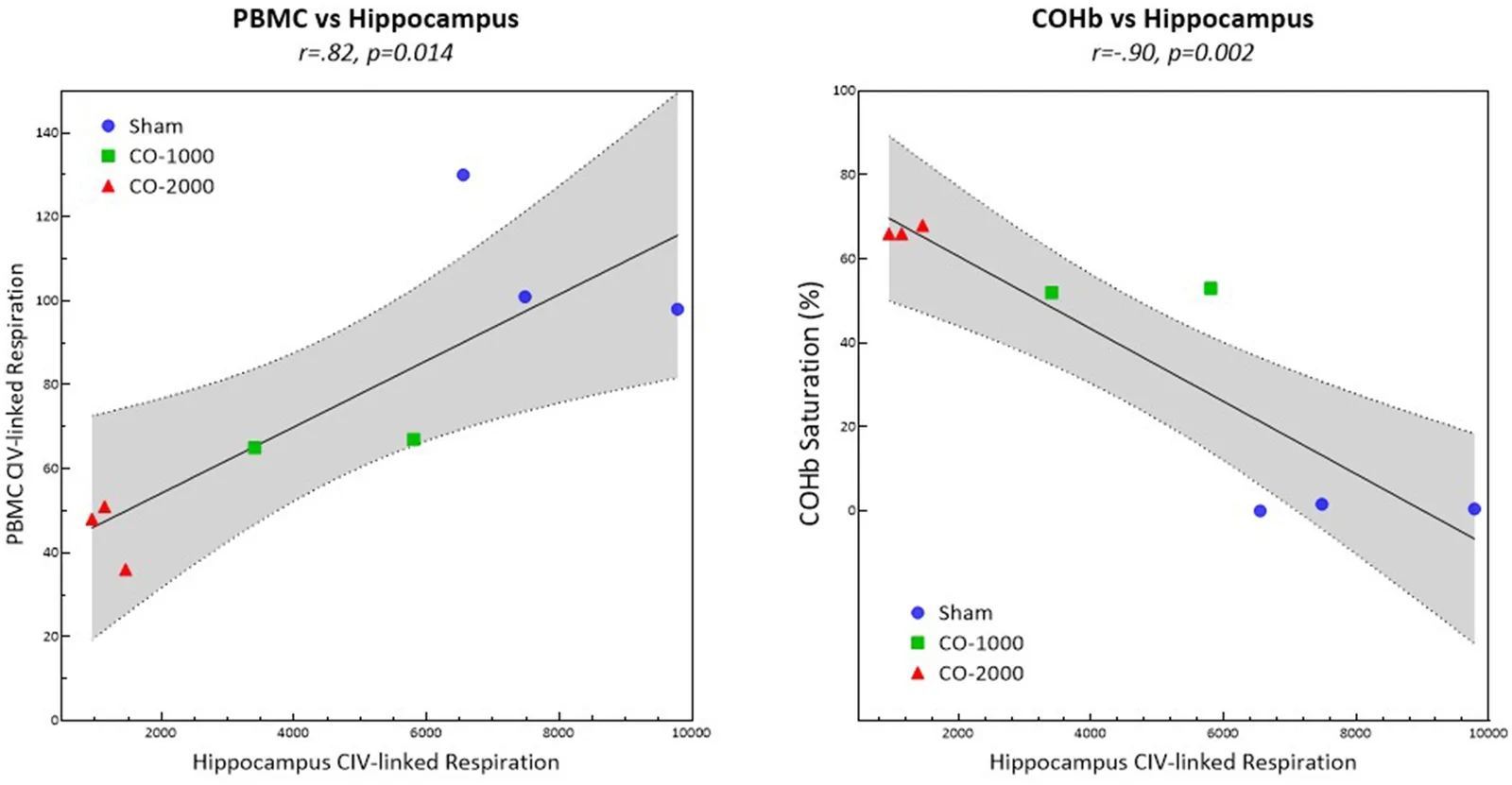
Correlation of PBMC CIV-linked respiration and COHb with hippocampal CIV-linked respiration. PBMC complex IV (CIV)-linked respiration demonstrated a significant positive correlation with hippocampal CIV-linked respiration (r = 0.82, *P* = 0.014), supporting the potential utility of PBMC mitochondrial bioenergetics as a minimally invasive systemic biomarker of cerebral mitochondrial dysfunction following acute carbon monoxide poisoning. COHb saturation demonstrated a significant inverse correlation with hippocampal CIV-linked respiration (r = −0.90, *P* = 0.002), consistent with increasing exposure severity being associated with worsening mitochondrial dysfunction. Due to limited PBMC sample availability and successful paired tissue acquisition, these analyses were performed in a subset of animals Sham (*n* = 3), CO-1000 (*n* = 2), and CO-2000 (*n* = 3) animals (total *N* = 8).

Paired PBMC and hippocampal mitochondrial respiration analyses were successfully obtained in a subset of animals across sham, CO-1000, and CO-2000 groups (Fig. 3). PBMC CIV-linked respiration demonstrated a significant positive correlation with hippocampal CIV-linked respiration, demonstrating a significant association between PBMC and hippocampal CIV-linked respiration across the experimental groups. These findings support the potential utility of PBMC mitochondrial respiration as an exploratory translational biomarker of cerebral bioenergetic dysfunction following acute CO exposure. Animals with lower PBMC CIV-linked respiration generally demonstrated more severe impairment in hippocampal respiratory capacity. COHb saturation demonstrated a significant inverse correlation with hippocampal CIV-linked respiration, indicating that greater CO exposure severity was associated with reduced mitochondrial respiratory function. However, PBMC respiration provides a direct functional bioenergetic measurement rather than an exposure burden metric alone.

### Cerebral microdialysis demonstrates dynamic metabolic disturbance during acute CO poisoning

**Fig. 4.**
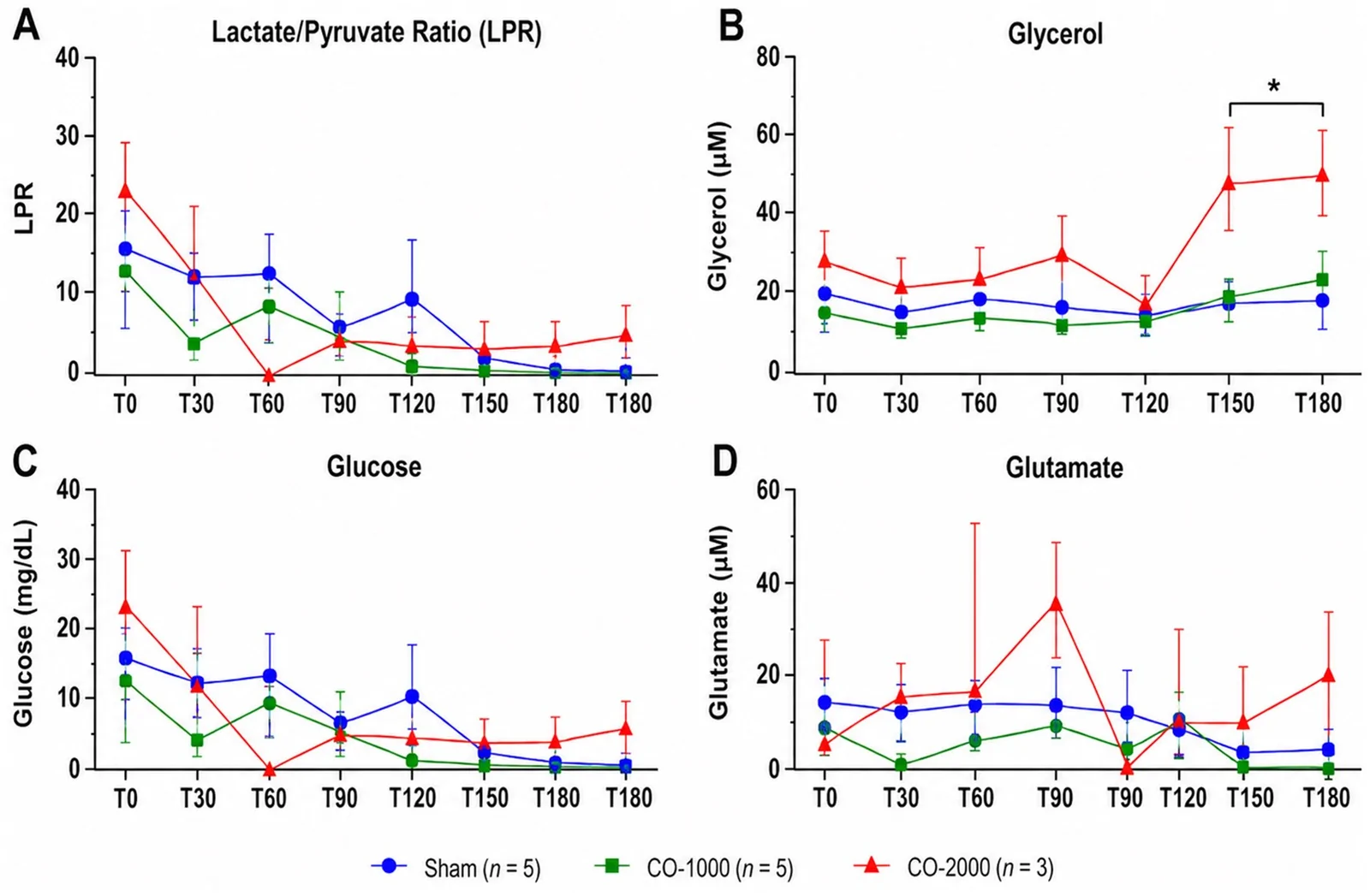
Cerebral microdialysis profiles during acute carbon monoxide poisoning in swine. Serial cerebral microdialysis was performed during CO exposure and recovery in sham, CO-1000, and CO-2000 animals. Panels show extracellular (A) lactate/pyruvate ratio (LPR), (B) glycerol, (C) glucose, and (D) glutamate concentrations over time. CO-exposed animals, particularly the CO-2000 group, demonstrated greater LPR variability and numerical elevations consistent with impaired oxidative metabolism and redox stress. Glycerol demonstrated the clearest exposure-associated separation, with delayed elevations in the CO-2000 group during late exposure/recovery, supporting increased cellular stress and membrane injury. Glucose and glutamate demonstrated dynamic but less robust group differences. These findings support dose-associated cerebral metabolic dysfunction during acute CO poisoning and complement ex vivo evidence of impaired mitochondrial respiration and reduced cortical ATP content. Data are presented as median with interquartile range. (Sham, *n* = 5; CO-1000, *n* = 5; CO-2000, *n* = 3). *P*-values for significant comparisons are indicated in the figure.

Cerebral microdialysis revealed time-dependent alterations in extracellular metabolites during carbon monoxide exposure and recovery (Fig. 4). The lactate/pyruvate ratio (LPR), a surrogate marker of impaired oxidative metabolism and altered cellular redox state, demonstrated temporal fluctuations throughout the experiment without significant differences between treatment groups or at individual time points (Fig. 4A). Although individual CO-exposed animals, particularly within the CO-2000 group, exhibited occasional elevations in LPR, substantial inter-animal variability precluded consistent group separation.

Extracellular glycerol, a marker of membrane phospholipid degradation and cellular injury, demonstrated the clearest group separation. Mixed-effects analysis identified significant effects of time (F(2.656, 25.23) = 5.719, *P *= 0.0052), exposure group (F(2, 11) = 11.28, *P *= 0.0022), and the time × exposure interaction (F(5.311, 25.23) = 3.451, *P *= 0.0150). Post hoc analysis demonstrated significantly higher extracellular glycerol concentrations in the CO-2000 group compared with sham animals at 150 min (adjusted *P *= 0.0416), consistent with greater membrane injury following severe carbon monoxide exposure. Microdialysate glucose changed significantly over time but did not differ significantly between treatment groups. Nevertheless, the CO-2000 group showed numerically higher glucose concentrations at later timepoints relative to sham and CO-1000 groups, potentially reflecting impaired cerebral glucose utilization during severe poisoning.

Extracellular glutamate demonstrated numerical elevations in the CO-2000 group at several intervals, although no significant main effects or interactions were detected. Collectively, the microdialysis profile supports dose-associated cerebral metabolic dysfunction during acute CO poisoning, with the strongest signals seen in markers of impaired oxidative metabolism and delayed cellular injury.

These in vivo findings were concordant with ex vivo bioenergetic analyses demonstrating reduced mitochondrial respiration—particularly within hippocampal tissue—and lower cortical ATP content in CO-exposed animals.

### Cytokine response

**Fig. 5.**
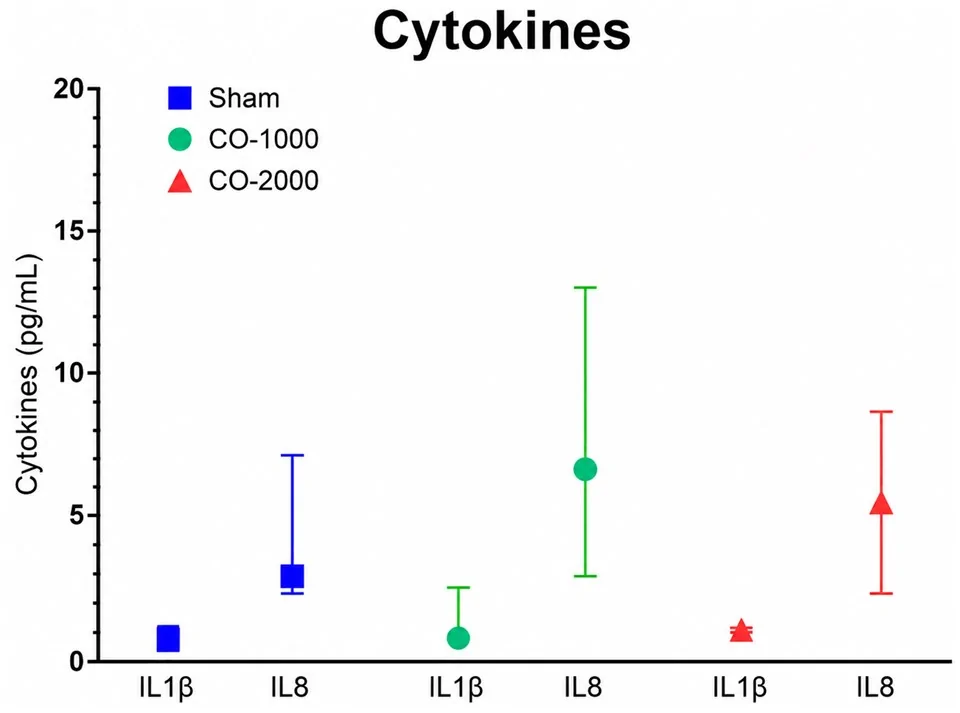
Terminal plasma cytokine concentrations following acute carbon monoxide exposure. Terminal plasma IL-1β (A) and IL-8 (B) concentrations were measured following sham, CO-1000, or CO-2000 exposure using a multiplex cytokine assay. Data are presented as individual animals with median and interquartile range. (Sham, *n* = 5; CO-1000, *n* = 5; CO-2000, *n* = 3). Extreme outlier values identified during assay quality review were excluded from analysis. No significant differences were detected among groups by one-way ANOVA. Modest numerical increases in IL-1β and IL-8 were observed in CO-exposed animals, although substantial inter-animal variability limited statistical separation.

Terminal plasma concentrations of IL-1β and IL-8 were measured following sham, CO-1000, and CO-2000 exposure (Fig. 5). After exclusion of extreme outlier values identified during quality review, no significant differences were observed between groups for either cytokine by one-way ANOVA. Terminal IL-1β concentrations demonstrated modest numerical increases in both CO exposure groups compared with sham. IL-8 concentrations were also numerically higher in the CO-1000 and CO-2000 groups relative to sham, although substantial inter-animal variability limited statistical separation. Overall, systemic cytokine responses were heterogeneous and less discriminative than the mitochondrial and bioenergetic abnormalities observed in this model.

### Western blot

**Fig. 6.**
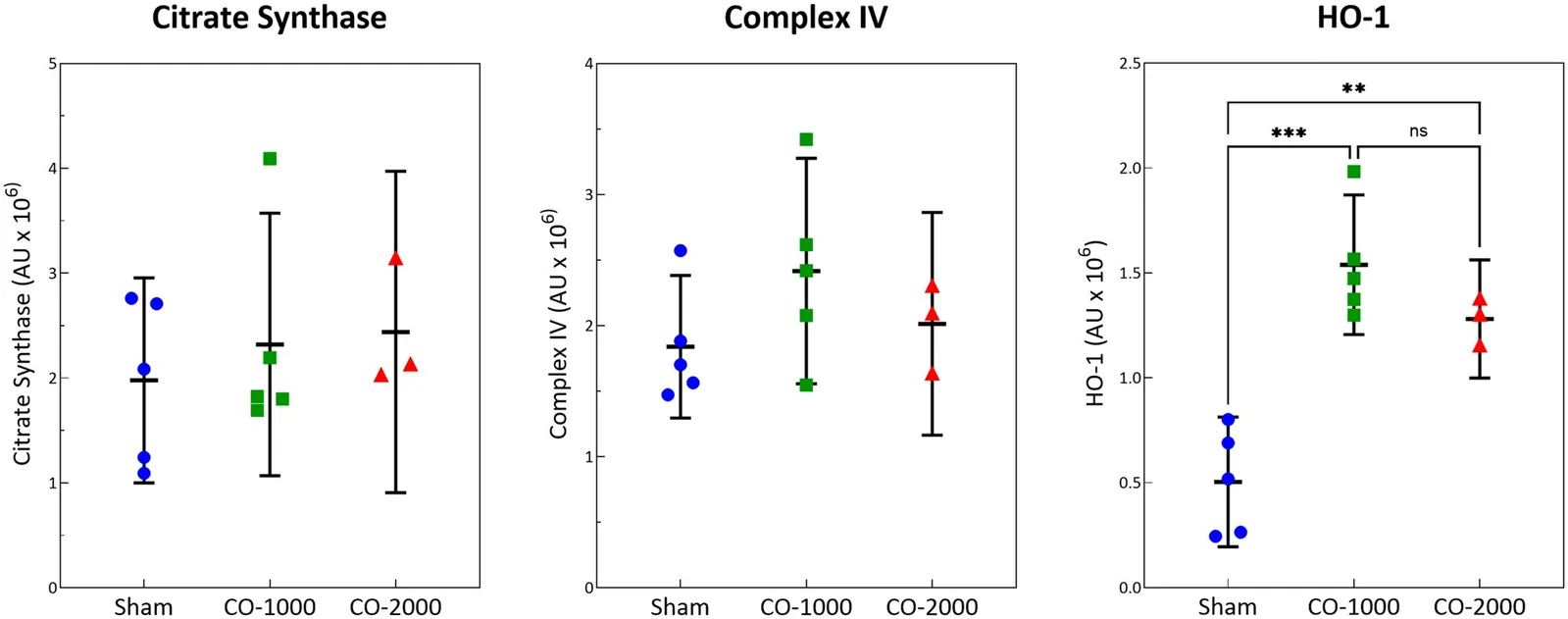
Cerebral mitochondrial and stress-response protein expression following acute carbon monoxide poisoning. Western blot densitometry of cerebral tissue harvested following acute CO exposure in sham, CO-1000 ppm, and CO-2000 ppm swine. Panels show (A) citrate synthase (CS), a marker of mitochondrial content; (B) Complex IV (COXIV) protein abundance; and (C) heme oxygenase-1 (HO-1), an inducible oxidative stress-response protein. Each data point represents one biological replicate. Technical replicate densitometry measurements obtained from duplicate lanes across two independent immunoblots were averaged prior to statistical analysis. Data are presented as mean ± SEM (Sham, *n *= 5; CO-1000, *n *= 5; CO-2000, *n *= 3). No significant group differences were observed for CS or Complex IV. HO-1 expression was significantly increased following CO exposure. Statistical significance is denoted as follows: Ns, not significant; *P *< 0.05 (*); *P* < 0.01 (**); *P* < 0.001 (***).

Western blot densitometry was performed to assess mitochondrial protein abundance and stress-response signaling in cerebral tissue following acute carbon monoxide exposure (Fig. 6). Representative Western blot images corresponding to the densitometric analyses are provided in Fig. S1. No significant differences in citrate synthase (CS) or Complex IV protein abundance were identified among sham, CO-1000 ppm, and CO-2000 ppm animals (one-way ANOVA, both *P *> 0.05). CS, used as a surrogate marker of mitochondrial content, remained unchanged across exposure groups. Similarly, Complex IV protein abundance did not differ significantly despite variability among individual animals.

In contrast, heme oxygenase-1 (HO-1) expression differed significantly among groups (one-way ANOVA, *P *< 0.001). Post hoc analysis demonstrated significantly increased HO-1 expression in both CO-exposed groups compared with sham (CO-1000 vs sham, *P *< 0.001; CO-2000 vs sham, *P *< 0.01), whereas HO-1 expression did not differ between the CO-1000 and CO-2000 groups. These findings indicate activation of oxidative stress-response pathways following acute carbon monoxide poisoning, even in the absence of measurable reductions in mitochondrial protein abundance. The dissociation between preserved CS and Complex IV protein expression and increased HO-1 expression suggests that acute carbon monoxide poisoning predominantly impairs mitochondrial function rather than mitochondrial content or respiratory chain protein abundance during the early postexposure period.

### Histopathology and immunohistochemistry

**Fig. 7.**
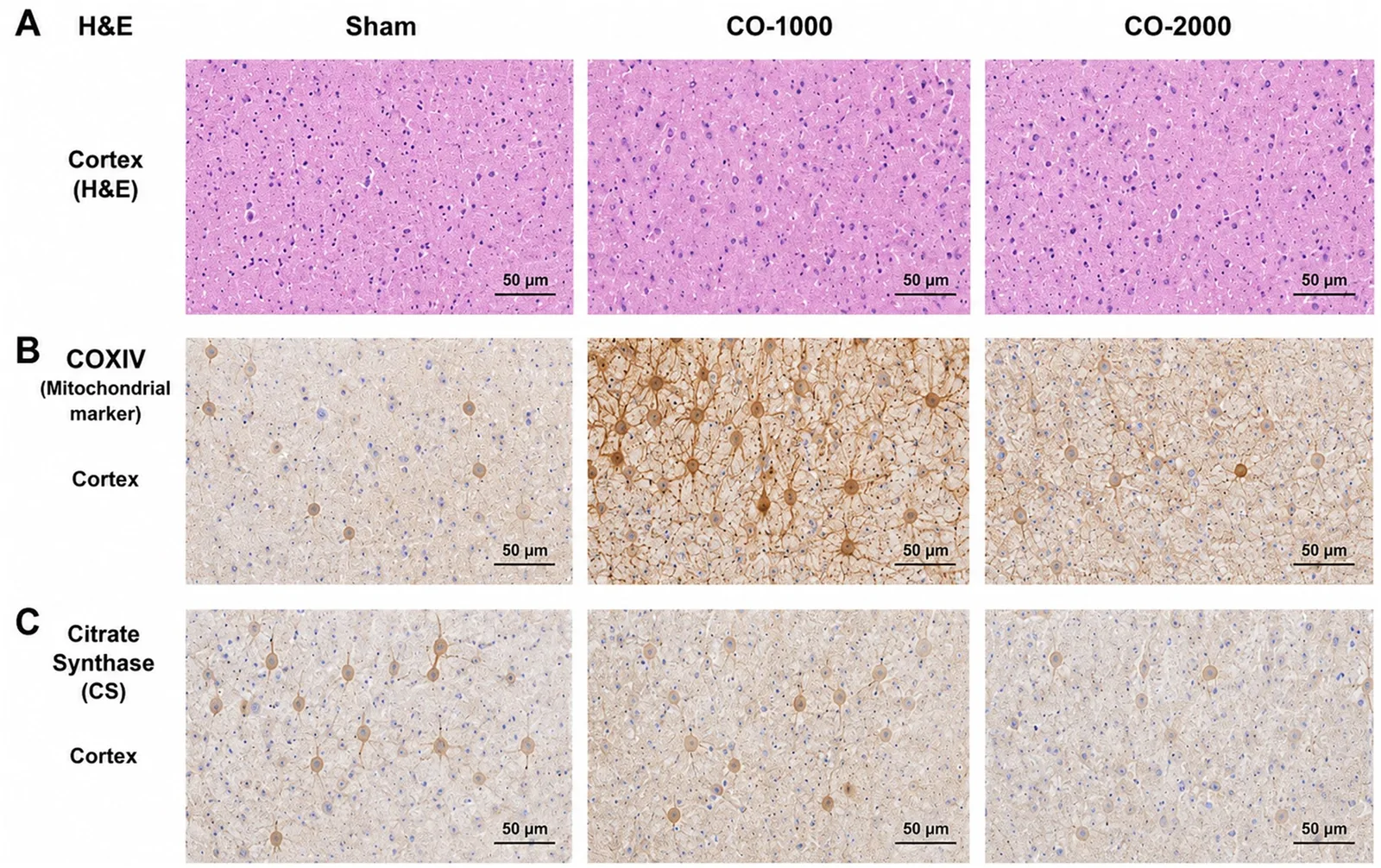
Representative histologic and immunohistochemical staining in cortical tissue following acute carbon monoxide exposure. Representative cortical sections from sham, CO-1000, and CO-2000 animals demonstrating (A) hematoxylin and eosin (H&E) staining, (B) Complex IV (COXIV) immunohistochemistry, and (C) citrate synthase (CS) immunohistochemistry. H&E staining demonstrates overall cortical tissue morphology, while COXIV and CS staining demonstrate mitochondrial protein expression and distribution. Representative images are shown to complement the quantitative protein analyses. Scale bars = 50 μm.

Histopathologic assessment was performed to evaluate structural injury and regional immunohistochemical staining patterns following acute carbon monoxide exposure (Fig. 7). Hematoxylin and eosin (H&E) staining demonstrated no overt histologic injury across sham, CO-1000 ppm, or CO-2000 ppm animals, with all sections receiving injury scores of 0.

Semiquantitative immunohistochemistry demonstrated generally mild and variable staining patterns across groups. Citrate synthase (CS) and Iba-1 staining remained minimal overall without a clear exposure-associated pattern, suggesting limited acute alterations in mitochondrial content or microglial activation at the time of tissue harvest. COXIV staining demonstrated the greatest degree of increased signal, with several CO-exposed animals exhibiting moderate-to-extensive staining increases relative to sham tissue. PARP staining remained minimal to mild overall. Representative PARP and Iba-1 immunohistochemical images are provided in Fig. S2.

Technical limitations include tissue folding, thick sectioning, and suboptimal staining quality limited interpretation of selected sections. Overall, these findings suggest that acute carbon monoxide exposure produced substantial functional metabolic and mitochondrial abnormalities in the absence of prominent early structural histopathologic injury.

## Discussion

Building upon our previously established translational swine model of carbon monoxide poisoning, the present study extends our previous work by providing a more comprehensive characterization of acute cerebral bioenergetic dysfunction through integrated physiologic, metabolic, mitochondrial, and histopathologic assessments. In this translational swine model of acute carbon monoxide poisoning, severe exposure produced dose-dependent systemic and cerebral metabolic dysfunction characterized by elevated lactate, impaired mitochondrial respiration, and reduced ATP content. Mitochondrial impairment was observed in both cortical and hippocampal tissue, with reductions across multiple respiratory states consistent with widespread cerebral bioenergetic dysfunction following acute CO exposure. These functional bioenergetic disturbances were detectable in the absence of overt histopathologic injury at the terminal timepoint, indicating that mitochondrial and metabolic dysfunction develop early during acute CO exposure. The acute, nonsurvivor design cannot establish whether these abnormalities resolve or progress to structural injury over time.

CO has traditionally been viewed primarily as a disorder of impaired oxygen delivery mediated through the formation of COHb (Ramirez et al. 1974; Jaffe 1997; Favory et al. 2006). Although this mechanism remains central to systemic toxicity, the current findings support a broader model in which direct mitochondrial dysfunction contributes substantially to cerebral injury. CO can bind cytochrome *c* oxidase (Complex IV) at the heme a3 site, potentially impairing oxidative phosphorylation and ATP generation, though the relative contribution of direct Complex IV inhibition versus hypoxia-mediated and postreoxygenation injury at clinically relevant COHb levels in vivo remains debated (Lopez et al. 2009; Akyol et al. 2014). In the present study, reductions in Complex IV-linked respiration were particularly evident within hippocampal tissue, providing functional evidence of persistent mitochondrial inhibition following acute exposure. Because Complex IV is critical for maintenance of the proton gradient required for ATP synthesis, dysfunction at this level likely contributes to downstream energetic failure, oxidative stress, and activation of cellular injury pathways.

One of the principal findings of this study was the demonstration of impaired mitochondrial respiration in both cortical and hippocampal tissue following acute carbon monoxide exposure. Although the pattern of affected respiratory states differed somewhat between brain regions, both cortex and hippocampus exhibited reductions in mitochondrial respiratory function, with Complex IV (CIV)-linked respiration emerging as one of the most consistently impaired respiratory states. These findings are mechanistically consistent with the well-established inhibitory effects of carbon monoxide on cytochrome c oxidase, the terminal enzyme of the electron transport chain (Lopez et al. 2009; Akyol et al. 2014). Impaired CIV activity would be expected to reduce electron transport, diminish oxidative phosphorylation, and ultimately contribute to ATP depletion and bioenergetic failure. The widespread suppression of mitochondrial respiration observed in both brain regions supports mitochondrial dysfunction as a central mechanism of acute CO neurotoxicity. Given the well-recognized association between carbon monoxide poisoning and delayed neurocognitive sequelae, including memory impairment and executive dysfunction, these findings provide mechanistic support linking early mitochondrial bioenergetic failure to subsequent neurologic injury (Chance and Williams 1955, 1956; Chance et al. 1955).

Although tissue ATP concentrations demonstrated a more pronounced dose-dependent decline than high-resolution mitochondrial respiration, these measures reflect complementary but distinct aspects of cerebral bioenergetic function. High-resolution respirometry assesses the maximal respiratory capacity of isolated mitochondria under controlled ex vivo substrate conditions, whereas tissue ATP concentrations represent the integrated in vivo energetic state and are influenced by mitochondrial function, oxygen delivery, substrate availability, cerebral perfusion, and cellular energy demand. Accordingly, mitochondrial respiratory dysfunction may occur at lower levels of CO exposure, whereas more severe exposure results in greater downstream energetic failure and ATP depletion. This interpretation suggests that ATP may serve as a more sensitive indicator of the cumulative physiologic consequences of severe CO poisoning, whereas high-resolution respirometry provides mechanistic insight into mitochondrial respiratory impairment.

Continuous cerebral microdialysis demonstrated dynamic metabolic alterations during acute carbon monoxide poisoning. Among the measured analytes, extracellular glycerol provided the strongest evidence of treatment-related cerebral injury, with delayed elevations observed following severe CO exposure. As a marker of membrane phospholipid degradation and cellular injury, increased glycerol is consistent with progressive structural cellular stress accompanying mitochondrial dysfunction. In contrast, lactate/pyruvate ratio (LPR), glucose, and glutamate exhibited temporal fluctuations throughout the experiment but did not demonstrate consistent treatment-related differences. Although occasional numerical elevations in LPR were observed in severely exposed animals, substantial inter-animal variability limited clear group discrimination. Collectively, these findings suggest that cerebral microdialysis captures dynamic metabolic responses to acute CO poisoning, with glycerol emerging as the most informative marker of delayed cellular injury in this experimental model. Glycerol elevations, particularly during the later exposure and recovery phases, suggest ongoing membrane phospholipid degradation and delayed cellular stress despite reoxygenation (Hara et al. 2004). Although glucose and glutamate changes were more variable, their directional trends remain biologically plausible and consistent with impaired cerebral energetics and excitotoxic signaling. The microdialysis findings aligned closely with the ex vivo mitochondrial respiration data and ATP measurements, supporting an integrated model of cerebral metabolic crisis and mitochondrial dysfunction following acute CO exposure.

The reduction in cortical ATP content observed in severely exposed animals provides additional evidence of impaired cerebral bioenergetics. ATP production is highly dependent on intact oxidative phosphorylation, and the observed reductions are biologically consistent with the mitochondrial respiratory suppression identified during Oroboros respirometry. Together, the respiration and ATP findings support energetic failure as a central downstream consequence of acute carbon monoxide toxicity. Interestingly, while mitochondrial respiration and ATP production were impaired, citrate synthase and Complex IV protein abundance were not significantly reduced. This suggests that acute CO poisoning produces functional inhibition of mitochondrial respiration rather than immediate loss of mitochondrial mass or depletion of respiratory protein content. By contrast, HO-1 expression was increased in exposed animals, likely reflecting oxidative stress and adaptive cellular signaling triggered by hypoxia, reoxygenation injury, and mitochondrial stress (Sjostrand 1949; Tenhunen et al. 1969; Clark et al. 2000). Although HO-1 generates endogenous carbon monoxide during heme degradation, its induction is generally interpreted as a protective stress-response pathway.

Oxidative stress has been widely implicated in the pathogenesis of carbon monoxide-induced neuronal injury through the generation of reactive oxygen species, lipid peroxidation, and mitochondrial damage. Although increased HO-1 expression observed in the present study is consistent with activation of a cellular stress response, direct markers of oxidative stress or antioxidant pathway activation were not evaluated. Consequently, the contribution of oxidative injury to the observed mitochondrial dysfunction cannot be determined from the current study and represents an important area for future investigation.

An important translational component of this study was the exploratory evaluation of peripheral blood mononuclear cell (PBMC) mitochondrial respiration as a systemic biomarker of cerebral mitochondrial dysfunction (Chacko et al. 2014, 2016). In a subset of animals with paired sampling, PBMC Complex IV-linked respiration correlated with hippocampal mitochondrial respiratory impairment, suggesting that peripheral blood bioenergetics may reflect tissue-level mitochondrial dysfunction following acute carbon monoxide exposure. Although COHb remains an important indicator of exposure severity, it does not directly assess downstream mitochondrial function or cellular energetic status. In contrast, PBMC mitochondrial respiration provides a minimally invasive and potentially serial biomarker of bioenergetic dysfunction (Jang et al. 2017a, 2017b, 2018a, 2019a; Acin-Perez et al. 2020). These findings remain preliminary given the limited sample size and incomplete paired sampling but support further investigation of PBMC bioenergetics as a translational monitoring tool in toxicologic and critical care settings. The observed correlation should be interpreted cautiously, as it was derived from pooled exposure groups and may have been influenced by the small number of surviving animals in the highest CO exposure group, which exhibited profound systemic physiologic compromise. Larger survivor studies will be necessary to determine whether PBMC mitochondrial respiration independently predicts cerebral mitochondrial dysfunction across a broader spectrum of injury severity.

Despite marked metabolic and mitochondrial dysfunction, overt structural injury was limited on histopathologic evaluation. H&E scores remained minimal across groups, whereas immunohistochemical changes were generally modest, with the clearest signal observed in COXIV staining. The relative absence of severe histologic injury despite substantial mitochondrial dysfunction suggests that bioenergetic failure and cerebral metabolic distress occur early in the injury process and may precede irreversible structural degeneration. This finding may have important therapeutic implications, as it suggests a potential treatment window during which mitochondrial-targeted interventions could mitigate downstream tissue injury before permanent structural damage develops (Singh et al. 2006).

Systemic cytokine responses were comparatively modest and variable. Although several cytokines demonstrated directional increases in exposed animals, statistically significant group differences were not consistently observed. These findings likely reflect the modest sample size, biologic variability, and relatively early terminal sampling window. It is also possible that circulating cytokines incompletely capture compartmentalized neuroinflammatory responses occurring within injured cerebral tissue (Holohan et al. 2008; Degrandi et al. 2009).

These findings have translational implications for the management of CO poisoning. Current therapeutic strategies primarily focus on restoration of oxygen delivery and displacement of CO from hemoglobin. However, the present data suggest that persistent mitochondrial dysfunction and cerebral metabolic failure may continue despite reoxygenation. Therapeutic strategies aimed at preserving mitochondrial respiration, stabilizing mitochondrial membranes, limiting oxidative stress, or restoring cellular energetics may therefore represent important adjunctive approaches. In addition, cerebral metabolic monitoring and peripheral bioenergetic biomarkers may provide clinically useful tools for assessing injury severity and treatment response beyond traditional exposure metrics such as COHb alone.

Several limitations should be acknowledged. This was a nonsurvivor model focused on acute injury and therefore does not address delayed neurologic recovery or chronic remodeling following exposure. Sample sizes were modest, and early mortality in the severe exposure group may have introduced survivorship bias in some analyses. In addition, isolated mitochondrial preparations provide high mechanistic resolution but may not fully recapitulate in vivo cellular interactions and compensatory responses. The PBMC analyses were exploratory and performed in only a subset of animals with successful paired sampling. Additionally, markers of oxidative stress and antioxidant pathway activation were not measured, limiting direct mechanistic assessment of oxidative injury following acute carbon monoxide exposure. Furthermore, because terminal tissue analyses required completion of the experimental protocol, measurements of mitochondrial respiration, tissue ATP, Western blot protein expression, and histopathology in the CO-2000 group necessarily reflect surviving animals and may underestimate the degree of cerebral bioenergetic dysfunction associated with the most severe CO exposure. Future studies should evaluate whether mitochondrial respiratory dysfunction correlates with long-term neurologic outcomes in survivor models and whether targeted mitochondrial therapies can restore bioenergetic function and reduce delayed neurologic injury. An additional limitation is that terminal tissue analyses required completion of the experimental protocol. Consequently, mitochondrial respiration, ATP, protein expression, and histopathologic measurements in the CO-2000 group were obtained only from animals surviving to the terminal timepoint, potentially underestimating the degree of cerebral bioenergetic dysfunction associated with the most severe exposure.

In conclusion, acute carbon monoxide poisoning in this translational swine model produced dose-dependent cerebral metabolic dysfunction characterized by impaired mitochondrial respiration, ATP depletion, oxidative stress signaling, and metabolic crisis, with particularly pronounced vulnerability within the hippocampus. These bioenergetic abnormalities were detectable in the absence of overt structural injury at the terminal timepoint, indicating that mitochondrial dysfunction is an early feature of acute CO toxicity; longitudinal survivor studies will be required to determine whether such dysfunction resolves or progresses to structural injury. These findings strengthen the emerging view that mitochondrial bioenergetic failure is a central component of acute CO neurotoxicity and support further investigation of mitochondrial-targeted therapies and translational bioenergetic biomarkers in carbon monoxide poisoning.

## Supplementary Material

kfag093_Supplementary_Data

## Data Availability

The data underlying this article will be made available by the corresponding author upon reasonable request.
