# Supplementary material for: Mitochondrial dysfunction, cerebral metabolic crisis, and exploratory bioenergetic biomarkers in a translational swine model of acute carbon monoxide poisoning
Source: Toxicol Sci. 2026 Jul 29;209(8):kfag093. doi: 10.1093/toxsci/kfag093 (PMC13503005; doi:10.1093/toxsci/kfag093)
Supplement: Supplementary file 1 [file kfag093_supplementary_data.zip › Supplementary Figure Legends.docx]

**Supplementary Figure Legends**

**Supplementary Figure S1 | Representative Western blot images corresponding to the cerebral protein expression analyses shown in Figure 6.** Representative immunoblots from sham, CO-1000 ppm, and CO-2000 ppm animals demonstrating cerebral protein expression of citrate synthase (CS), Complex IV (COXIV), and heme oxygenase-1 (HO-1). Corresponding loading controls (GAPDH for CS and GA1R for COXIV) are shown. These representative blots correspond to the densitometric analyses presented in Figure 6**.**

**Supplementary Figure S2| Representative immunohistochemical staining of cortical tissue following acute carbon monoxide exposure.** Representative cortical sections from sham, CO-1000, and CO-2000 animals demonstrating (A) hematoxylin and eosin (H&E) staining, (B) COXIV immunohistochemistry as a mitochondrial marker, and (C) citrate synthase (CS) immunohistochemistry. H&E staining demonstrated no overt acute structural injury at the terminal 150-minute timepoint. COXIV staining demonstrated increased mitochondrial-associated staining in CO-exposed animals, while CS staining remained mild and variable without a clear exposure-dependent reduction. Scale bars = 50 µm.
